# Environmental Factors That Contribute to the Maintenance of *Cryptococcus neoformans* Pathogenesis

**DOI:** 10.3390/microorganisms8020180

**Published:** 2020-01-28

**Authors:** Maphori Maliehe, Mathope A. Ntoi, Shayanki Lahiri, Olufemi S. Folorunso, Adepemi O. Ogundeji, Carolina H. Pohl, Olihile M. Sebolai

**Affiliations:** Department of Microbial, Biochemical and Food Biotechnology, University of the Free State, Bloemfontein 9301, Free State, South Africa; maphorimaliehe1@gmail.com (M.M.); mathope.ntoi@gmail.com (M.A.N.); shayanki22@gmail.com (S.L.); 2014061341@ufs4life.ac.za (O.S.F.); OgundejiAO@ufs.ac.za (A.O.O.); pohlch@ufs.ac.za (C.H.P.)

**Keywords:** *Cryptococcus*, environmental factors, pathogenesis, survival, virulence factors, transcriptional factors, signalling factors

## Abstract

The ability of microorganisms to colonise and display an intracellular lifestyle within a host body increases their fitness to survive and avoid extinction. This host–pathogen association drives microbial evolution, as such organisms are under selective pressure and can become more pathogenic. Some of these microorganisms can quickly spread through the environment via transmission. The non-transmittable fungal pathogens, such as *Cryptococcus,* probably return into the environment upon decomposition of the infected host. This review analyses whether re-entry of the pathogen into the environment causes restoration of its non-pathogenic state or whether environmental factors and parameters assist them in maintaining pathogenesis. *Cryptococcus (C.) neoformans* is therefore used as a model organism to evaluate the impact of environmental stress factors that aid the survival and pathogenesis of *C. neoformans* intracellularly and extracellularly.

## 1. Introduction

The genus *Cryptococcus* is defined by basidiomycetous fungi that can transform into a yeast state [1]. This polyphyletic genus is home to over 50 species; however, two of them have emerged as important human pathogens [2]. *Cryptococcus (C.) neoformans*, one of these pathogenic species, was first described from a surgical specimen of a 31-year-old woman in 1894 by pathologist Otto Busse. In the same year, Sanfelice isolated the same yeast from peach juice and named it *Saccharomyces neoformans*, but because the organism neither ferments nor forms ascospores, Vuillemin renamed it as *C. neoformans* in 1901 [3].

In general, when cultivated on 2% malt agar, *C. neoformans* forms smooth, mucoid cream coloured colonies [4]. Furthermore, when examined under the microscope, the yeast appears as encapsulated, globose to ovoid-shaped cells with a diameter between 2.5 and 10 μm. Initially, isolates were grouped according to their capsular epitopes and further into varieties based on molecular typing [5,6,7,8,9,10,11]. Since then, *C. neoformans* and the closely related species, *C. gattii*, have undergone numerous nomenclature revisions. The most recent system proposes a group of seven species, with those previously designated *C. neoformans* var. *grubii*, now regarded as *C. neoformans sensu lato* and those designated *C. neoformans* var. *neoformans*, now elevated to species level and designated as *C. deneoformans* (which is endemic in Europe) [12].

At the beginning of the 1900s, this organism was considered a rare species. However, a gradual increase in the population presenting with immunocompromised conditions due to human immunodeficiency virus (HIV) infections and the use of immunosuppressive therapies led to a concomitant rise in isolation of *C. neoformans* in the clinical setting [13]. *C. neoformans* is ubiquitous in the environment and is frequently found in soil contaminated with bird droppings [14]. Globally, cryptococcal cells have been isolated from their most common environmental habitats: trees such as *Eucalyptus* [15].

When desiccated yeast cells or basidiospores are inhaled, they can lodge in the alveoli of hosts—despite airway turbulence in the respiratory tract [16]—leading to the development of primary lung infection [2]. In immunocompetent individuals, the immune system is usually able to clear the invading fungal cells from the body [17]. However, if the immune system is impaired, the cells can disseminate via a haematogenous route (sometimes by hiding inside macrophages) to practically every organ in the body, including the central nervous system (CNS) [18]. Interestingly, cryptococcal cells have been shown to have a particular predilection for the brain. Following the successful crossing of the blood–brain barrier (BBB), the fungal cells hinder the brain’s ability to reabsorb cerebrospinal fluid (CSF), consequently causing an inflammatory condition called meningoencephalitis [19]. This condition is often fatal in immunocompromised cases and is also regarded as an acquired immunodeficiency syndrome (AIDS)-defining illness [20]. Thus, it is not surprising to document high mortality rates due to CNS cryptococcal infection among people infected with HIV [21].

## 2. Survival of *C. neoformans* in the Environment

Some microorganisms can spread from person-to-person through direct transmission, e.g., *Mycobacterium tuberculosis*, which spreads through cough-generated aerosols [22]. Fortunately, in the case of *C. neoformans*, the organism is unable to disseminate from an infected person to a healthy individual, limiting the spread of the fungus between people [23]. A plausible way for cryptococcal cells to return to the environment is via the decomposition of an infected host or host body part. Taking into consideration a large number of microbes that populate environmental niches, and the challenges and threats they must confront to survive [24,25], it is logical that organisms must have individualised survival strategies to negotiate the changes in environments and stimulants. To adequately respond to these changes, various signalling processes are initiated in the fungus that allows for the development, growth, and production of virulence factors. Close to 1700 genes have been identified to be responsible for environmental stress response (ESR) in *C. neoformans* [26,27,28]. There is a biochemical relay of signals that are involved in this adaptation: the sensors/receptors/adaptors, G-proteins, secondary messengers, protein kinases, transcriptional factors, and regulators [29,30].

**Figure 1:**
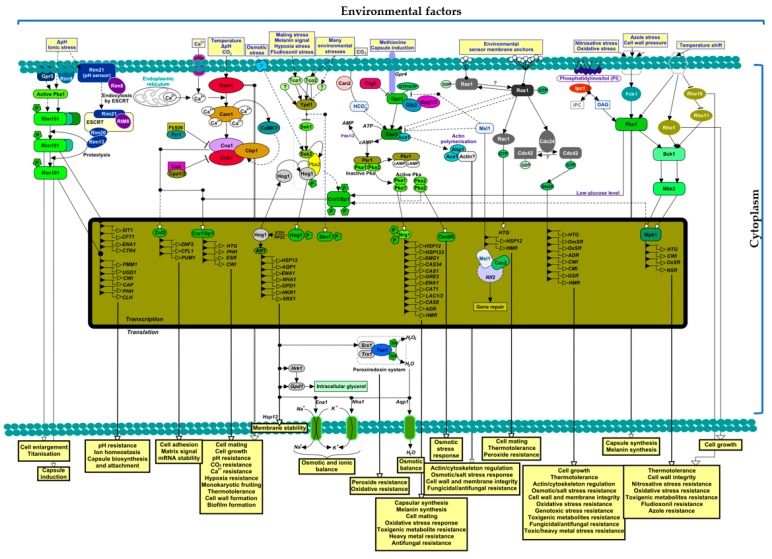
The interplay of different signalling pathways that enable *C. neoformans* to turn environmental stresses into phenotypic and physiologic advantages that help in the production of virulence factors during pathogenesis in the host. A number of pathways are indicated above. The Grp5–Rim101 is a Pka-dependent transcriptional factor that undergoes possibly sequential and enhanced proteolysis after activation. This pathway is utilised to enlarge the cell size, maintain homeostasis, and synthesis capsules. The Ca^2+^–Calmodulin–Calcineurin pathway that may utilise Ca^2+^ to initiate several transcriptional factors through the activation of *Znf2* or *Crz1/Sp1*. This pathway promotes multi-resistance, cell growth, and biofilm formation. The Tco–Ypd1–Ssk1//Ssk2–Pbs2–Hog1–Crz/Sp1 phosphorylation pathways are phosphorelay cascade systems that can sense many environmental factors and enhance virulence production through the activation of different transcriptional factors that enable membrane stability, osmotic balance, and several exogenous resistances. The Hog1 pathway can also regulate the capsule, melanin, and ergosterol synthesis. The Gpr4–Gpa–Cac1–cAMP–Pka can sense exogenous methionine during mating and capsule induction. The downstream of this pathway is regulated by *Pkr1* while the transcriptional factors are under the influence of activated *Ngr1*. In a yet unknown way, the presence of CO_2_ through *Can2* can also activate this pathway; however, *Msl1*, which seemed to be involved in the chromatin assembly factor (CAF-1) for DNA repair may be repressing this pathway. The Ras1–Rac1//Ras1–Cdc24–Cdc42–Ste20 is sensitive to several environmental factors, and together with cAMP–Pka enhances virulence production such as capsule, melanin, actin, and several cellular resistances and tolerances. The Pdk1–Pkc1–Bck1–Mkk2–Mpk1 can be activated by metabolic stress, phosphatidylinositol (PI), and Rho-family proteins. Activated Pkc can phosphorylate several intracellular messengers, including *Crz/Sp1*, and initiate capsule and melanin production. Several transcriptional factors for resistance and tolerance can also be initiated by the *Mpk1* complemented by the *Rho*-family proteins. LEGEND: Δ—change; dashed line arrows—possible positive regulation/activation; solid line arrows—activation/production/initiation/organelle membrane crossing; dashed white line arrows—possible stimulation; solid line black arrows—stimulation; thin/double-thin line white arrows—nucleus-independent stimulation; dashed/solid/thin line bar—repression/inhibition/decrease; ?—unknown.

As shown in Figure 1, *C. neoformans* uses the high-osmolarity glycerol (HOG) response pathway to resist diverse environmental stresses (such as osmotic shock, oxidative stress, nutrient shortage, and genotoxic stress) and express some phenotypic traits in growth, differentiation, production of virulence factors, and ergosterol biosynthesis [31]. This HOG pathway consists of mitogen-activated protein kinase (MAPK) and Hog1 and subsequently Pbs2 MAPK kinase (MAPKK) and Ssk2/22 MAPKK kinase (MAPKKK). These pathways are stress-activated to increase the phosphorylation of Hog1 to resist various environmental stresses [32]. The HOG pathway controls other subsets of pathways and the expression of other regulatory genes. These include cation transporters (*ENA1*) and pH homeostatic genes (*NHA1*) that are co-regulated by pH-sensing Rim101 and Nrg1 signalling pathways [31,33], aquaporin gene (*AQP1*) for water balance, sulfiredoxin gene (*SRX1*) against peroxides [31,34], and signalling regulator kinases such as Hrk1 [35] and Sch9 kinases [31] for osmotic balance and oxidative stress, respectively.

Furthermore, the HOG pathway regulates the transcriptional factors, such as Atf1, for sensing diverse environmental factors and regulating virulence factors [36,37] and Mbs1, for genotoxic stress response, ergosterol biosynthesis, membrane integrity, oxidative stress response, osmotic stress response, and the production of the virulence factors [38]. There are yet unknown upstream molecules that may control the activation of Ssk2–Pbs2–Hog1 cascade events (Figure 1).

Moreover, *C. neoformans* harnesses secondary messengers to regulate cellular signalling pathways. cAMP/PKA (cyclic AMP/protein kinase A) has emerged as a core messenger involved in cryptococcal pathogenesis [39,40,41,42,43]. Together with the Ras signalling pathway, cAMP/PKA independently regulates and controls the *C. neoformans* responses to the environmental stresses. It has been reported that Ras and its downstream signalling factors such as Rac1 and its paralogues are more critical for *C. neoformans* to adapt to osmotic pressure, thermotolerance, actin polymerisation, and cell wall formation than cAMP/PKA [36,44,45,46]. However, components of cAMP signals such as Aca1, Pka1, and Pka2 seem to play subordinate roles in adaptation to environmental stresses [28] and are not involved in hyphae formation in *C. neoformans* during sensing and adaptation [47]. Furthermore, independent of the cAMP/PKA, Aca1 has been shown to complement the efforts of Ras for adequate diverse stress response in *C. neoformans* [48]. Nevertheless, the signal components (Cac1, Aca1, Gpa1, Pka1, and Pka2) of cAMP/PKA are exclusively crucial for resistance to environmental oxidants, heavy metals, and toxigenic compounds [28] (Figure 1).

Apart from these key signalling pathways, *C. neoformans* also uses other related pathways and virulence proteins to suppress environmental stress. Msl1-like protein, which negatively regulates cAMP signalling upstream of Cac1, produces phenotypic changes that are independent of Ras and cAMP/PKA leading to thermotolerance, peroxide resistance, gene repair, and cell mating [49,50] (Figure 1). Sch9 kinase Hog1-dependent signalling pathway, which is a nutrient-sensing signalling pathway like the TORC1 pathway [51], promotes cellular adaptation to oxidative and thermal stresses [31,52,53]. However, this pathway can sometimes bypass the Hog1 regulatory mechanism to facilitate environmental stress response to toxigenic heavy metals, cations, and antifungals [53]. *C. neoformans* can combine the Rim101–PKA dependent signalling pathways to sense nutrient and mineral depletion [54,55], adapt to pH changes in the macrophage phagolysosome, CSF, and serum [56,57]. The same pathways are responsible for cell wall remodelling [56] and titanisation [58].

Ca^2+^-dependent/independent-Calmodulin and Calcineurin-dependent/independent-Calmodulin signalling pathways in *C. neoformans* are involved in thermotolerance, high CO_2_ resistance, alkaline pH resistance, cell wall integrity and architecture, and cell mating [59,60,61,62,63,64,65]. Lastly, Pkc1 and Mpk1 MAPK signalling pathways are involved in azole and toxigenic resistance by controlling the sphingolipid level [32,66], cell wall integrity [67], stress tolerance [68], thermotolerance [69], and oxidative and nitrosative tolerance [70] (Figure 1). Thus, it is clear that *C. neoformans* evolved mechanisms to transform environmental stress signals into an enabled phenotypic and metabolic status that promotes cryptococcal pathogenesis.

## 3. Environmental Factors that Assist in the Maintenance of Virulence Traits

### 3.1. Thermotolerance

Temperature shift is an important ecological factor that microbes ought to successfully negotiate. Cryptococcal cells are mesophilic, and thus well adapted to survive at lower environmental temperatures than in mammalian bodies. Hence cryptococcal cells have been isolated from environmental hosts such as amoeba [71]. Given the vast difference in the internal temperature of a host such as an amoeba compared to a mammalian host, it is clear the argument of Guijarro and co-workers is true that the regulation of virulence genes in response to temperature shift is modulated in different ways depending on the hosts [72].

This saprophytic organism has been isolated from trees where metabolic energy is derived by decomposing components of the wood via the action of laccase enzyme [73]. Laccases are widely distributed in nature and catalyse a number of important functional processes such as lignification of plant walls as well as the production of melanin [74]. Thus, while this enzyme assists cryptococcal cells in accessing food in the environment, it has, at the same time, been shown to be co-opted to perform other roles in the physiology of this organism [74]. To the point, the laccase enzyme can be used to produce melanin from phenolic substrates found in the environment [75]. Production of melanin is reported to aid cells during the daytime to withstand ultraviolet (UV) light [76] by capturing radiation energy from the sun and converting it to chemical energy [77]. This quality (melanisation) also helps other organisms to harvest thermal radiation in vents found in the deep, dark ocean [77] or in the case of cryptococcal cells, when colonising the “dark” spaces of mammalian host bodies [78].

To prevent environmental microbes from colonising mammalian bodies, mammals have a higher internal temperature; however, this came at a considerable energy cost [79]. The benefit of this is the fundamental limitation it places on microbes as their membranes begin to lose selective permeability, and protein becomes denatured at 37 °C [80,81]. Therefore, to survive and operate optimally at a higher temperature, microbes ought to undergo some molecular reorganisation [82]. For example, cells can deploy heat shock protein 90 (hsp90) that is critical in thermotolerance [83]. *Hsp90* is a molecular chaperone that is universal in all eukaryotic organisms and is responsible for regulating the morphology of *C. neoformans* at high temperatures [84,85]. This protein is documented to be expressed at low levels at 25 °C to suppress the expression of heat shock transcriptional factor (HSF) [86]. At this temperature, this protein shows limited protein-folding capabilities, but rather assists in regulating the aggregation of already-folded proteins [87]. It has been shown that a temperature change from 25 to 37 °C results in the upregulation of hsp90 in cryptococcal cells [88]. Studies have shown that the inhibition of this gene results in minimal growth at 37 °C and, thus, is critical for growth at high temperatures [89]. A perfect organism that illustrates the importance of thermotolerance is *C. podzolicus*, which, despite having essential virulence factors such as the capsule and melanin, is non-infectious because it cannot grow at 37 °C [90].

The ability of *C. neoformans* to produce different types of hsps is pivotal to its survival in mammalian hosts. Several hsps (60, 70, and 80) have been identified as prominent antigens in animals and humans infected with *C. neoformans* [91,92]. However, the *hsp12* family seemed not to be affected by temperature change [93]. The elevated levels of proteases such as carboxypeptidase D, a serine protease, hydroxylases, and phenolic metabolic enzymes, are a reminiscence of the importance of melanin production at 37 °C during *C. neoformans* infections [78,92].

Generally, *C. neoformans* adapts to high temperatures by the stress-sensing signalling pathway (Figure 1) and elevating transcriptional responses for the hsp, translational machinery, mitochondria accessory proteins, and stress response proteins (such as superoxide dismutase and peroxidase). In the works of [44,59], it was reported that mutations in the *RAS1* and *CNA1* genes of *C. neoformans* produced avirulent and growth defective strains at 37 °C. Using serial analysis of gene expression (SAGE) and ribonucleic acid (RNA) blotting, an average of 12.5% differential transcription has been observed in *C. neoformans* (serotype A strain H99) when cultivated at 25 and 37 °C [92]. Further analysis showed that ribosomal proteins, translational elongation factor-1α and initiation factor, cyclophilin A (CPA1 and CPA2), thioredoxin peroxidase, and superoxide dismutase are more recruited at 37 °C [92,94]. However, zinc transport protein is the most highly expressed tag at 25 °C [92].

Furthermore, Wang and co-workers in 2001 identified Cpa1 as one of the single transcriptional factor required by *C. neoformans* to grow at elevated temperatures and to produce virulence factors [95]. Proteins such as endoplasmic reticulum (ER) chaperone binding immunoglobulin protein (BiP), benzodiazepine receptor homolog, and several ribosomal proteins are exclusively produced at 37 °C [96]; while transcripts for histones H1, H3, H4, and translational elongation factor-3 (TEF-3) are more predominantly found at 25 °C [92]. It has been shown that H1 and H4 are differentially regulated based on the temperature. Elevated temperature introduces conformational changes into the genes, which can alter the relative expression of histone family proteins hence, *C. neoformans* can swiftly produce H4 at 37 °C to re-establish the core histones anchor for chromatin stability, but H1 is more produced at 25 °C to maximise the chromatin packages within the nucleus [92].

The cell membrane is critical as it also provides a platform, from which receptor proteins can initiate signalling processes [97,98]. Therefore, maintaining the stability of these important cellular components at higher temperatures is critical. The latter can be achieved through the upregulation of enzymes involved in sterol and lipid metabolism. Steen and co-workers in 2002 revealed an elevated level of sterol oxidase and fatty acid desaturase and synthase at 25 °C in *C. neoformans*, which enables the pathogen to produce more unsaturated fatty acids to be incorporated into the membrane phospholipids for adequate membrane fluidity and integrity [92]. Moreover, *C. neoformans* massively transcribed genes involved in transportation and assimilation of nutrients at 25 °C during filamentation and sporulation, and these include zinc transporter, iron permease, glucose transporters, inositol transporter, and inositol synthase [92]. These proteins are essential for the survival of this pathogen in the host central nervous system (CNS) and brain, which are rich in glucose and inositol [99,100]. There is strong speculation that the cAMP pathway may be responsible for this upregulation because of its role in the *C. neoformans* virulence production [47,101].

An important quality of cryptococcal cells is the ability to undergo a morphological change. The dimorphic nature of the organism also allows diploid strains to grow vegetatively as yeasts at 37 °C and to display a filamentous morphology at 24 °C [102]. The latter form can further degenerate into haploid spores at a lower temperature [102].

### 3.2. pH Tolerance

Birds, especially pigeons, are considered as reservoirs of cryptococcal cells, and they account for the geographical spread of the fungus [103]. These birds re-introduce the cryptococcal cells into the environment by releasing their droppings into the soil [104]. The bird droppings lower the pH of the contaminated soil (due to the presence of uric acid in the excreta), which can favour the replication of the cryptococcal cells [16]. Cryptococcal cells form capsules for protection against harsh conditions through the upregulation of highly conserved pH-response regulatory genes [18]. Therefore, it is not surprising to observe environmental isolates with marked capsule formation [105]. However, pH can also fluctuate and impact growth. Thus, the ability to adapt to changes in pH is critical for survival in the environment and host cells.

Although cryptococcal cells often disseminate via a haematogenous route wherein the pH of the blood is neutral, they have also been found in phagosomes and guts of birds, wherein the pH is generally acidic. This (acidic environment), however, does not seem to inhibit the growth of these cells, which suggests that *C. neoformans* is well adapted to low pH [57]. In part, this adaptation to acidic pH is beneficial to cryptococcal cells in that it allows them access to iron [106,107], which is inaccessible at neutral pH due to iron being bound to transferrin [108].

Phagocytic cells can acidify their phagolysosomal compartments, which is an essential antimicrobial mechanism during infection by pathogenic agents [58]. However, Leon-Rodriguez and co-workers showed that cryptococcal cells could modulate the pH levels in these compartments, resulting in their incomplete acidification [109]. Additionally, Fu and co-workers showed that by deploying ureases, cells could increase the pH, allowing them to survive for longer periods inside the macrophages [110]. This pH modulation occurs because of urea hydrolysis to yield ammonia, which is toxic to host cells and might damage the host tissue to promote transmigration [111], and possibly their escape from macrophages.

### 3.3. Tolerance to the Limitation of Nutrients and Water

Upon re-entry into the soil contaminated with bird droppings; a niche characterised by low temperature and pH, cells also find themselves in proximity to other organisms [112] and often, they engage in antagonistic interactions to appropriate nutrients. The yeast must derive energy for cellular processes as well as obtain space to accommodate its expanding population. During the competition, yeast cells often experience nutrient and water limitation [113]. The latter may cause yeast cells to become desiccated. This aids with survival during this period by reducing the metabolic activity of the cells. Limited metabolic activity results in the conservation of energy, therefore allowing the cell to survive for a longer period. It has been observed that *Cryptococcus* can remain alive for more than two years without nutrition and water in the environmental sources, such as dried pigeon guano [114]. The removal of water from the cells also results in a lighter weight, which aids effective short- and long-distance dispersion to other niches with more nutrients [115]. This phenomenon, therefore, increases the chances of cell persistence in the environment.

Lee et al. documented that nutrient limitation such as nitrogen deficiency and lower glucose concentration in media can result in a morphological switch to pseudohyphae in both *C. neoformans* and *C. gattii* [116]. However, this change may be reversible or irreversible in some [116]. *C. neoformans* cells in nutrient limiting conditions produce hyphae, basidia, and spore chains by bisexual and unisexual reproduction. The hyphal structure enables the cells to explore the environment, bringing them into contact with mating partners as well as to forage for nutrient sources [117].

During bisexual reproduction opposite mating type cells secrete pheromones under nutrient-limiting conditions, and a compatible mating type would sense these molecules (secreted by the opposite mating type) via the pheromone MAPK signalling circuit [118], initiating the formation of conjugation tubes leading to cell–cell fusion. The two cells form a diploid heterokaryon and initiate filamentous growth. Basidia are formed apically at the filament where nuclear fusion and meiosis occur, and several rounds of mitosis produce chains of basidiospores [119,120]. These spores are carried externally on the basidium, which facilitates ease of air dispersion. The swept-off spores can begin to colonise new niches of the soil (including mammalian bodies) with sufficient nutrients.

On the contrary, Hsueh et al. mention in their study, that there are G-protein-dependent signalling pathways that influence sexual mating in *C. neoformans* serotype D, which involves three G protein α subunits, Gpa1, Gpa2, and Gpa3. During nutrient-limiting conditions, serotype D cells express Gpa3 to inhibit basal signalling required for filamentous growth and, therefore, prevent the induction of pheromone response. On pheromone activation, Gpa2 is expressed, contributing to the pheromone response that leads to mating [121].

An essential aspect of sexual reproduction is the elimination of deleterious mutations leading to a fit progeny with genetic diversity [120]. *C. neoformans* consists of two mating types, i.e., MAT**a** and MAT**α**. However, the majority of environmental and clinical isolates are MATα, which is reported to be associated with more severe infections in HIV-infected persons [122]. The frequency of the MAT**α** suggests it may confer an environmental and clinical advantage. Genetic studies have also shown that clinical isolates are more diverse and include the isolation of the rare environmental mating-type, MAT**a** [103,123,124].

### 3.4. Predation Selects for Resistance to Phagocytosis

Cryptococcal cells can also fall prey to holozoic organisms such as amoeba [125]. Amoebaepredate on organisms such as *C. neoformans* via receptor-mediated phagocytosis. In brief, the process entails the recognition of the target cell’s microbe-associated molecular patterns (found on the cell surface) by the predator’s pattern recognition receptors, followed by actin polymerisation, which facilitates the movement and extension of pseudopods to capture the targeted cell. The target cell is then internalised and trapped within the food vacuole. The harsh environment that prevails inside this compartment is sufficient to kill an internalised cell under normal physiological conditions [126,127]. The killing is facilitated by oxygen-dependent and oxygen-independent mechanisms. In the oxygen-dependent mechanism, the lumen is flooded with reactive oxygen species that target the macromolecules of the internalised cell. In the oxygen-independent mechanism, the lumen of the food vacuole is acidified and is inundated with antimicrobial peptides such as amoeba-pore and acanthaporin, among others [126,128,129]. These peptides kill the engulfed cells by creating pores in their cell wall [130].

The fact that amoebae can predate on other organisms to support its growth comes with evolutionary consequences. With the exertion of sufficient predatory pressure, prey develops microbial factor(s) to subvert or circumvent the deleterious effects of the pressure. Therefore, through this interaction, cryptococcal cells, for example, become trained to better deal with other phagocytic cells such as macrophages. Interestingly, it has been theorised that phagocytic cells such as macrophages may have evolved from the free-living amoebae [125].

Phagocytosis is a process that evolved a long time ago and is exquisitely effective in enabling phagocyte-like amoeba to obtain nutrients. Thus, from their interaction with these predators, cryptococcal cells are well sensitised to the threat of being captured and internalised and have developed several defence mechanisms that protect them from phagocytosis by both amoeba and macrophages.

Capsular components of *Cryptococcus* are known to bind and prevent antibody and complement binding [131]. Glucuronoxylomannan (GXM), the major component of the cryptococcal polysaccharide capsule, has been shown to modulate internalisation of cells by phagocytic cells [132]. In their study, Madu and co-workers showed that GXM decreased the levels of a fetuin-like molecule in amoeba [133]. This protein has been reported to mediate the uptake of particulate material such as bacteria and fragments of apoptotic cells by the phagocytes [134,135]. Therefore, reduced levels of fetuin may account for cryptococcal cells escaping capture and internalisation. In the same study, it was shown that 3-hydroxy fatty acids also inhibited the production of fetuin [133]. Unlike the GXM, 3-hydroxy fatty acids are only transiently associated with capsules during their release into the extracellular environment [136]. It is conceivable that GXM and capsular 3-hydroxy fatty acids may also affect the levels of fetuin in a mammalian host to avoid being phagocytosed because, as argued by Steenbergen and Casadevall—cryptococcal cells may perceive all phagocytic cells as the same [137]. In the main, when cultivated, cryptococcal cells display a uniform cell wall structure. However, exposure to certain cues may see them express morphological plasticity. For example, when threatened by environmental predator such as amoeba, a cryptococcal cell may transition to form an enlarged cell, and a similar transformation may occur when attacked by immune cells due to shared similarities between amoebae and macrophages. Based on the latter, it is conceivable that the cue(threat) ought to be the same, regardless of the environment they arise from, in order for the displayed behaviour (morphological–functional characteristics) to be consistent, i.e. confer protection [137]. However, not all cryptococcal cells in the host transition to form titan cells. In this case, it is possible that the prevailing environmental factors that surround a cryptococcal cell may also contribute to lack of titanisation.

In the host environment, several factors such as high CO_2_, iron limitation, oxidative stress, and pH changes may induce enlargement of cells (titanisation) as a mechanism of protecting them from these stressful conditions [138]. However, this comes at a high energy cost and is highly dependent on mitochondrial activity [139] to support the complex re-wiring of the metabolism [140,141]. These larger cells are not easily internalised as they exceed the phagocytes in size [142]. Therefore, the ability of cells to enlarge enhances their virulence further. Studies performed by Granger and co-workers showed that there is a direct correlation between cell size and decreased phagocytosis by alveolar macrophages [143]. Moreover, it is reported that elements of the pheromone MAPK signalling circuit in mating-type MATa cells also regulate titan cell formation [58]. Although titan cell formation is initially advantageous to cryptococcal cells regarding phagocytosis avoidance in the lungs, it does interfere later with dissemination to the brain [144].

Phagosome-containing cryptococcal cells were shown to be “leaky”, aiding in the intracellular survival and replication of *C. neoformans* [145]. The leakage allows vacuoles containing GXM from the capsule to escape into the cytoplasm of phagocytic cells and interfere with some glycolytic enzymes thereby resulting in a loss of acidity and allows capsular cells to harness nutrients [146]. Once this occurs, the phagocytic cells may lose their integrity and rupture, or they may allow the cryptococcal cells to exit, furthering their dissemination in the host [147].

For phagocytosis to be initiated, complement receptors may be required to participate [148]. These receptors are integrins and have been found on a host of phagocytic cells [149]. These proteins recognise several endogenous ligands, including a specific sugar at the cell membrane of *C. neoformans*, which they can tag. Interestingly, integrins are also present in holozoic organisms [150]. In their report, Cornillon and co-workers speculated that these proteins could be products of convergent evolution due to shared features [150]. Thus, cryptococcal cells may produce an anti-phagocytic molecule called the anti-phagocytic protein 1 to avoid being tagged by environmental predators [151]. In mammals, the anti-phagocytic protein 1 is reported to bind complement 2, and 3 factors and in turn prevent the recognition of iC3b fragments on the cryptococcal cells by the complement 3 opsonic receptor, CR3 [151]. Other studies have shown that this protein is upregulated in glucose-limiting conditions and high temperatures (37 °C) [152].

Macrophages and polymorphonuclear neutrophil cells create oxidative stress by producing reactive oxygen species (ROS), in the form of OH^−^ and O^2−^, which are important in killing cells trapped inside the phagosomes to prevent further dissemination [153]. Here, these radicals can kill internalised microbes directly via oxidative damage to cellular macromolecules or indirectly by stimulating non-oxidative mechanisms such as attracting phagocytes [154]. Despite this, there is evidence in some infections, that participation of radicals may not be sufficient to reduce the microbial burden. To circumvent the challenge of being subjected to oxidative stress, microbes may scavenge or neutralise radicals.

Several microbial antioxidant enzymes have been implicated in resolving ROS produced by host immune cells. For example, H_2_O_2_ can be decomposed by enzymes such as catalases, catalase-peroxidases, peroxidases, glutathione peroxidases and the glutathione system, peroxiredoxins, and the thioredoxin system [155]. ROS produced externally or during normal metabolism (usually to prevent fungal infection) has generated considerable interest in defining the components of the antioxidant response and studying their role as virulence determinants in fungi such as *C. neoformans*. In *Cryptococcus*, the expression of the *SOD1* gene increases at the physiological temperature (37 °C). It has been observed that a *SOD1* mutant lacking the cytosolic Cu- and Zn-SOD is killed by ROS in a cell-free system and is significantly less virulent than the wild-type strain in a murine infection model [156].

Moreover, mitochondrial SOD2p is a crucial factor for the survival of *C. neoformans* and *C. gattii* at 37 °C, to cope with different stress conditions and is essential to cause infection in a murine model of cryptococcosis. Strains lacking both SOD1p and SOD2p were found to be even more susceptible to oxidative and other types of stress, and incapable of producing the experimental disease [156]. The results of an investigation showing that mice deficient in neutrophil serine proteases, but normal in ROS production, are still susceptible to fungal infection establishes a less direct role of the ROS generated by the macrophages in the microbial killing.

Internalised pathogens also encounter a nitrosative stress in the form of reactive nitrogen species (RNS) consisting of the free radical nitric oxide (NO) as well as products derived from NO such as nitrite, nitrogen dioxide, and nitrate or from the reaction of NO with other molecules to generate peroxynitrite, peroxynitrous acid, or nitrosothiols [157]. There are only preliminary level studies that attempted to establish the genetic basis of cryptococcal resistance towards nitrosative stress [157]. For example, Naslund and co-workers reported the internalisation of cryptococcal cells within the macrophage cell line did not directly induce the production of nitric oxide synthase—suggesting there may be enzyme repression [158]. It is also possible that radicals may be quenched as microbial enzymes such as flavohemoglobin denitrosylase have been implicated in the direct consumption of NO [158]. The deletion of this enzyme in *C. neoformans* was shown to attenuate this organism’s virulence in a murine model [159].

Cryptococcal cells release melanin in multiple oxidation and reduction steps [160]. For example, a phenolic compound such as 3,4 dihydroxyphenylalanine (DOPA) can be converted to dopaquinone in a rate-limiting step catalysed by phenoloxidase with subsequent steps in the pathway leading to the production of melanin [75]. Melanin is an effective scavenger of free radicals. The proposed mechanism by which it protects against antioxidants is by its charged groups in the melanin polymer [161]. In addition to its role in melanin synthesis, studies have shown that laccases can produce microbial-oxygenated lipids such as prostaglandins and leukotrienes [162]. These lipids are important immunomodulators that promote the progression of the infection.

## 4. Conclusions

*C. neoformans* does not require a host such as a mammal to complete its life cycle. However, its condition in the environment and interaction with other organisms, including predators, has allowed evolution and maintenance of virulence traits that are critical to its survival inside the mammalian phagocytic cells and, hence, a successful mammalian pathogen. Its ubiquitous nature in the environment means that it has interacted with several organisms, including phagocytic predators such as amoeba. This constant interaction specifically has resulted in the acquisition of virulence factors that enhance the natural survival of the cryptococcal cells. The concerted efforts of these acquired virulence factors enabled this fungus to circumvent and elude the mammalian immune response during infection, leading to the latency and persistence of cryptococcal infection. A number of these virulence factors have been extensively studied, and therefore, with this knowledge, more effective ways to control infections can be developed. One avenue that can be explored is the disruption (using chemical analogues) of the underlying signalling pathways that are critical for the downstream changes in gene expression. These compounds could be used alone or as an adjunct to boost the efficacy of the current antimicrobial agents.

## References

[1] Idnurm A. (2010). A tetrad analysis of the basidiomycete fungus Cryptococcus neoformans. Genetics.

[2] Maziarz E.K., Perfect J.R. (2016). Cryptococcosis. Infect. Dis. Clin. N. Am..

[3] Srikanta D., Santiago-Tirado F.H., Doering T.L. (2014). Cryptococcus neoformans: Historical curiosity to modern pathogen. Yeast.

[4] Kwon-Chung K.J. (2011). Filobasidiella Kwon-Chung. The Yeasts.

[5] Varma A., Kwon-Chung K.J. (1992). DNA probe for strain typing of Cryptococcus neoformans. J. Clin. Microbiol..

[6] Crampin A.C., Matthews R.C., Hall D., Evans E.G.V. (1993). PCR fingerprinting Cryptococcus neoformans by random amplification of polymorphic DNA. J. Med. Vet. Mycol..

[7] Currie B.P., Freundlich L.F., Casadevall A. (1994). Restriction fragment length polymorphism analysis of Cryptococcus neoformans isolates from environmental (pigeon excreta) and clinical sources in New York City. J. Clin. Microbiol..

[8] Spitzer S.G., Spitzer E.D. (1994). Characterization of the CNRE-1 family of repetitive DNA elements in Cryptococcus neoformans. Gene.

[9] Brandt M.E., Hutwagner L.C., Kuykendall R.J., Pinner R.W. (1995). Comparison of multilocus enzyme electrophoresis and random amplified polymorphic DNA analysis for molecular subtyping of Cryptococcus neoformans. The Cryplococcal Disease Active Surveillance Group. J. Clin. Microbiol..

[10] Meyer W., Marszewska K., Amirmostofian M., Igreja R.P., Hardtke C., Methling K., Viviani M.A., Chindamporn A., Sukroongreung S., John M.A. (1999). Molecular typing of global isolates of Cryptococcus neoformans var. neoformans by polymerase chain reaction fingerprinting and randomly amplified polymorphic DNA-a pilot study to standardize techniques on which to base a detailed epidemiological survey. Electrophoresis.

[11] Meyer W., Castañeda A., Jackson S., Huynh M., Castañeda E. (2003). Molecular typing of IberoAmerican Cryptococcus neoformans isolates. Emerg. Infect. Dis..

[12] Hagen F., Khayhan K., Theelen B., Kolecka A., Polacheck I., Sionov E., Falk R., Parnmen S., Lumbsch H.T., Boekhout T. (2015). Recognition of seven species in the Cryptococcus gattii/Cryptococcus neoformans species complex. Fungal Genet. Biol..

[13] Kwon-Chung K.J., Fraser J.A., Doering T.L., Wang Z., Janbon G., Idnurm A., Bahn Y.-S. (2014). Cryptococcus neoformans and Cryptococcus gattii, the etiologic agents of cryptococcosis. Cold Spring Harb. Perspect. Med..

[14] Esher S.K., Zaragoza O., Alspaugh J.A. (2018). Cryptococcal pathogenic mechanisms: A dangerous trip from the environment to the brain. Mem. Inst. Oswaldo Cruz.

[15] Elhariri M., Hamza D., Elhelw R., Refai M. (2016). Eucalyptus Tree: A Potential Source of Cryptococcus neoformans in Egyptian Environment. Int. J. Microbiol..

[16] Chae H.S., Jang G.E., Kim N.H., Son H.R., Lee J.H., Kim S.H., Park G.N., Jo H.J., Kim J.T., Chang K.S. (2012). Classification of Cryptococcus neoformans and yeast-like fungus isolates from pigeon droppings by colony phenotyping and ITS genotyping and their seasonal variations in Korea. Avian Dis..

[17] Voelz K., May R.C. (2010). Cryptococcal interactions with the host immune system. Eukaryot. Cell.

[18] May R.C., Stone N.R.H., Wiesner D.L., Bicanic T., Nielsen K. (2016). Cryptococcus: From environmental saprophyte to global pathogen. Nat. Rev. Microbiol..

[19] O’Meara T.R., Alspaugh J.A. (2012). The Cryptococcus neoformans capsule: A sword and a shield. Clin. Microbiol. Rev..

[20] Nweze E.I., Kechia F.A., Dibua U.E., Eze C., Onoja U.S. (2015). Isolation of Cryptococcus neoformans from environmental samples collected in Southeastern Nigeria. Rev. Inst. Med. Trop. Sao Paulo.

[21] Sloan D., Parris V. (2014). Cryptococcal meningitis: Epidemiology and therapeutic options. CLEP.

[22] Shiloh M.U. (2016). Mechanisms of mycobacterial transmission: How does Mycobacterium tuberculosis enter and escape from the human host. Future Microbiol..

[23] Olsen S.J., Campbell A.P., Supawat K., Liamsuwan S., Chotpitayasunondh T., Laptikulthum S., Viriyavejakul A., Tantirittisak T., Tunlayadechanont S., Visudtibhan A. (2015). Infectious causes of encephalitis and meningoencephalitis in Thailand, 2003–2005. Emerg. Infect. Dis..

[24] Haruta S., Kanno N. (2015). Survivability of Microbes in Natural Environments and Their Ecological Impacts. Microbes Environ..

[25] Bauer M.A., Kainz K., Carmona-Gutierrez D., Madeo F. (2018). Microbial wars: Competition in ecological niches and within the microbiome. Microb. Cell.

[26] Cramer K.L., Gerrald Q.D., Nichols C.B., Price M.S., Alspaugh J.A. (2006). Transcription factor Nrg1 mediates capsule formation, stress response, and pathogenesis in Cryptococcus neoformans. Eukaryot. Cell.

[27] Hu G., Steen B.R., Lian T., Sham A.P., Tam N., Tangen K.L., Kronstad J.W. (2007). Transcriptional regulation by protein kinase a in Cryptococcus neoformans. PLoS Pathog..

[28] Maeng S., Ko Y.-J., Kim G.-B., Jung K.-W., Floyd A., Heitman J., Bahn Y.-S. (2010). Comparative transcriptome analysis reveals novel roles of the Ras and cyclic AMP signaling pathways in environmental stress response and antifungal drug sensitivity in Cryptococcus neoformans. Eukaryot. Cell.

[29] Bahn Y.-S., Kojima K., Cox G.M., Heitman J. (2006). A unique fungal two-component system regulates stress responses, drug sensitivity, sexual development, and virulence of Cryptococcus neoformans. Mol. Biol. Cell.

[30] Bahn Y.-S. (2008). Master and commander in fungal pathogens: The two-component system and the HOG signaling pathway. Eukaryot. Cell.

[31] Ko Y.-J., Yu Y.M., Kim G.-B., Lee G.-W., Maeng P.J., Kim S., Floyd A., Heitman J., Bahn Y.-S. (2009). Remodeling of global transcription patterns of Cryptococcus neoformans genes mediated by the stress-activated HOG signaling pathways. Eukaryot. Cell.

[32] Bahn Y.-S., Geunes-Boyer S., Heitman J. (2007). Ssk2 mitogen-activated protein kinase kinase kinase governs divergent patterns of the stress-activated Hog1 signaling pathway in Cryptococcus neoformans. Eukaryot. Cell.

[33] Idnurm A., Walton F.J., Floyd A., Reedy J.L., Heitman J. (2009). Identification of ENA1 as a virulence gene of the human pathogenic fungus Cryptococcus neoformans through signature-tagged insertional mutagenesis. Eukaryot. Cell.

[34] Soriano F.X., Baxter P., Murray L.M., Sporn M.B., Gillingwater T.H., Hardingham G.E. (2009). Transcriptional regulation of the AP-1 and Nrf2 target gene sulfiredoxin. Mol. Cells.

[35] Kim S.-Y., Ko Y.-J., Jung K.-W., Strain A., Nielsen K., Bahn Y.-S. (2011). Hrk1 plays both Hog1-dependent and -independent roles in controlling stress response and antifungal drug resistance in Cryptococcus neoformans. PLoS ONE.

[36] Kim M.S., Ko Y.-J., Maeng S., Floyd A., Heitman J., Bahn Y.-S. (2010). Comparative transcriptome analysis of the CO_2_ sensing pathway via differential expression of carbonic anhydrase in Cryptococcus neoformans. Genetics.

[37] Missall T.A., Lodge J.K. (2005). Function of the thioredoxin proteins in Cryptococcus neoformans during stress or virulence and regulation by putative transcriptional modulators. Mol. Microbiol..

[38] Song M.-H., Lee J.-W., Kim M.S., Yoon J.-K., White T.C., Floyd A., Heitman J., Strain A.K., Nielsen J.N., Nielsen K. (2012). A flucytosine-responsive Mbp1/Swi4-like protein, Mbs1, plays pleiotropic roles in antifungal drug resistance, stress response, and virulence of Cryptococcus neoformans. Eukaryot. Cell.

[39] Alspaugh J.A., Perfect J.R., Heitman J. (1998). Signal Transduction Pathways Regulating Differentiation and Pathogenicity ofCryptococcus neoformans. Fungal Genet. Biol..

[40] Pukkila-Worley R., Alspaugh J.A. (2004). Cyclic AMP signaling in Cryptococcus neoformans. FEMS Yeast Res..

[41] Bahn Y.-S., Xue C., Idnurm A., Rutherford J.C., Heitman J., Cardenas M.E. (2007). Sensing the environment: Lessons from fungi. Nat. Rev. Microbiol..

[42] Kozubowski L., Lee S.C., Heitman J. (2009). Signalling pathways in the pathogenesis of Cryptococcus. Cell. Microbiol..

[43] Kronstad J.W., Hu G., Choi J. (2011). The cAMP/Protein Kinase A Pathway and Virulence in Cryptococcus neoformans. Mycobiology.

[44] Alspaugh J.A., Cavallo L.M., Perfect J.R., Heitman J. (2000). RAS1 regulates filamentation, mating and growth at high temperature of Cryptococcus neoformans. Mol. Microbiol..

[45] Vallim M.A., Nichols C.B., Fernandes L., Cramer K.L., Alspaugh J.A. (2005). A Rac homolog functions downstream of Ras1 to control hyphal differentiation and high-temperature growth in the pathogenic fungus Cryptococcus neoformans. Eukaryot. Cell.

[46] Nichols C.B., Perfect Z.H., Alspaugh J.A. (2007). A Ras1-Cdc24 signal transduction pathway mediates thermotolerance in the fungal pathogen Cryptococcus neoformans. Mol. Microbiol..

[47] Alspaugh J.A., Pukkila-Worley R., Harashima T., Cavallo L.M., Funnell D., Cox G.M., Perfect J.R., Kronstad J.W., Heitman J. (2002). Adenylyl cyclase functions downstream of the Galpha protein Gpa1 and controls mating and pathogenicity of Cryptococcus neoformans. Eukaryot. Cell.

[48] Bahn Y.-S., Hicks J.K., Giles S.S., Cox G.M., Heitman J. (2004). Adenylyl cyclase-associated protein Aca1 regulates virulence and differentiation of Cryptococcus neoformans via the cyclic AMP-protein kinase A cascade. Eukaryot. Cell.

[49] Yang D.-H., Maeng S., Strain A.K., Floyd A., Nielsen K., Heitman J., Bahn Y.-S. (2012). Pleiotropic roles of the Msi1-like protein Msl1 in Cryptococcus neoformans. Eukaryot. Cell.

[50] Yang D.-H., Maeng S., Bahn Y.-S. (2013). Msi1-Like (MSIL) Proteins in Fungi. Mycobiology.

[51] Urban J., Soulard A., Huber A., Lippman S., Mukhopadhyay D., Deloche O., Wanke V., Anrather D., Ammerer G., Riezman H. (2007). Sch9 is a major target of TORC1 in Saccharomyces cerevisiae. Mol. Cell.

[52] Wang P., Cox G.M., Heitman J. (2004). A Sch9 protein kinase homologue controlling virulence independently of the cAMP pathway in Cryptococcus neoformans. Curr. Genet..

[53] Kim M.S., Kim S.-Y., Yoon J.K., Lee Y.-W., Bahn Y.-S. (2009). An efficient gene-disruption method in Cryptococcus neoformans by double-joint PCR with NAT-split markers. Biochem. Biophys. Res. Commun..

[54] Ding C., Yin J., Tovar E.M.M., Fitzpatrick D.A., Higgins D.G., Thiele D.J. (2011). The copper regulon of the human fungal pathogen Cryptococcus neoformans H99. Mol. Microbiol..

[55] Ding C., Festa R.A., Chen Y.-L., Espart A., Palacios Ò., Espín J., Capdevila M., Atrian S., Heitman J., Thiele D.J. (2013). Cryptococcus neoformans copper detoxification machinery is critical for fungal virulence. Cell Host Microbe.

[56] O’Meara T.R., Holmer S.M., Selvig K., Dietrich F., Alspaugh J.A. (2013). Cryptococcus neoformans Rim101 is associated with cell wall remodeling and evasion of the host immune responses. MBio.

[57] O’Meara T.R., Norton D., Price M.S., Hay C., Clements M.F., Nichols C.B., Alspaugh J.A. (2010). Interaction of Cryptococcus neoformans Rim101 and protein kinase A regulates capsule. PLoS Pathog..

[58] Okagaki L.H., Wang Y., Ballou E.R., O’Meara T.R., Bahn Y.-S., Alspaugh J.A., Xue C., Nielsen K. (2011). Cryptococcal titan cell formation is regulated by G-protein signaling in response to multiple stimuli. Eukaryot. Cell.

[59] Odom A., Muir S., Lim E., Toffaletti D.L., Perfect J., Heitman J. (1997). Calcineurin is required for virulence of Cryptococcus neoformans. EMBO J..

[60] Görlach J., Fox D.S., Cutler N.S., Cox G.M., Perfect J.R., Heitman J. (2000). Identification and characterization of a highly conserved calcineurin binding protein, CBP1/calcipressin, in Cryptococcus neoformans. EMBO J..

[61] Fox D.S., Cruz M.C., Sia R.A., Ke H., Cox G.M., Cardenas M.E., Heitman J. (2001). Calcineurin regulatory subunit is essential for virulence and mediates interactions with FKBP12-FK506 in Cryptococcus neoformans. Mol. Microbiol..

[62] Kraus P.R., Heitman J. (2003). Coping with stress: Calmodulin and calcineurin in model and pathogenic fungi. Biochem. Biophys. Res. Commun..

[63] Kraus P.R., Nichols C.B., Heitman J. (2005). Calcium- and calcineurinindependent roles for calmodulin in Cryptococcus neoformans morphogenesis and high-temperature growth. Eukaryot. Cell.

[64] Brown S.M., Campbell L.T., Lodge J.K. (2007). Cryptococcus neoformans, a fungus under stress. Curr. Opin. Microbiol..

[65] Steinbach W.J., Reedy J.L., Cramer R.A., Perfect J.R., Heitman J. (2007). Harnessing calcineurin as a novel anti-infective agent against invasive fungal infections. Nat. Rev. Microbiol..

[66] Lee H., Khanal Lamichhane A., Garraffo H.M., Kwon-Chung K.J., Chang Y.C. (2012). Involvement of PDK1, PKC and TOR signalling pathways in basal fluconazole tolerance in Cryptococcus neoformans. Mol. Microbiol..

[67] Gerik K.J., Donlin M.J., Soto C.E., Banks A.M., Banks I.R., Maligie M.A., Selitrennikoff C.P., Lodge J.K. (2005). Cell wall integrity is dependent on the PKC1 signal transduction pathway in Cryptococcus neoformans. Mol. Microbiol..

[68] Chabrier-Roselló Y., Gerik K.J., Koselny K., DiDone L., Lodge J.K., Krysan D.J. (2013). Cryptococcus neoformans phosphoinositide-dependent kinase 1 (PDK1) ortholog is required for stress tolerance and survival in murine phagocytes. Eukaryot. Cell.

[69] Lam W.C., Gerik K.J., Lodge J.K. (2013). Role of Cryptococcus neoformans Rho1 GTPases in the PKC1 signaling pathway in response to thermal stress. Eukaryot. Cell.

[70] Gerik K.J., Bhimireddy S.R., Ryerse J.S., Specht C.A., Lodge J.K. (2008). PKC1 is essential for protection against both oxidative and nitrosative stresses, cell integrity, and normal manifestation of virulence factors in the pathogenic fungus Cryptococcus neoformans. Eukaryot. Cell.

[71] Watkins R.A., King J.S., Johnston S.A. (2017). Nutritional Requirements and Their Importance for Virulence of Pathogenic Cryptococcus Species. Microorganisms.

[72] Guijarro J.A., Cascales D., García-Torrico A.I., García-Domínguez M., Méndez J. (2015). Temperature-dependent expression of virulence genes in fish-pathogenic bacteria. Front. Microbiol..

[73] Williamson P.R. (1997). Laccase and melanin in the pathogenesis of Cryptococcus neoformans. Front. Biosci..

[74] Zhu X., Williamson P.R. (2004). Role of laccase in the biology and virulence of Cryptococcus neoformans. FEMS Yeast Res..

[75] Buchanan K.L., Murphy J.W. (1998). What makes Cryptococcus neoformans a pathogen?. Emerg. Infect. Dis..

[76] Gerstein A.C., Neilson K. (2017). It’s not all about us: Evolution and maintenance of Cryptococcus virulence requires selection outside the human host. Yeast.

[77] Dadachova E., Bryan R.A., Huang X., Moadel T., Schwetzer A.D., Aisen P., Nosanchuk J.D., Casadevall A. (2007). Ionizing radiation changes the electronic properties of melanin and enhances the growth of melanized fungi. PLoS ONE.

[78] Casadevall A., Perfect J.R. (1998). Cryptococcus Neoformans.

[79] Casadevall A. (2016). Thermal Restriction as an Antimicrobial Function of Fever. PLoS Pathog..

[80] Leach M.D., Cowen L.E. (2013). Surviving the heat of the moment: A fungal pathogens perspective. PLoS Pathog..

[81] Ianutsevich E.A., Danilova O.A., Groza N.V., Kotlova E.R., Tereshina V.M. (2016). Heat shock response of thermophilic fungi: Membrane lipids and soluble carbohydrates under elevated temperatures. Microbiology (Read. Engl.).

[82] Gasch A.P., Spellman P.T., Kao C.M., Carmel-Harel O., Eisen M.B., Storz G., Botstein D., Brown P.O. (2000). Genomic expression programs in the response of yeast cells to environmental changes. Mol. Biol. Cell.

[83] Kozeko L.G. (2014). Changes in heat shock protein synthesis and thermotolerance of Arabidopsis thaliana seedlings as a result of inhibition of Hsp90 by geldanamycin. Tsitologiia.

[84] Leach M.D., Klipp E., Cowen L.E., Brown A.J.P. (2012). Fungal Hsp90: A biological transistor that tunes cellular outputs to thermal inputs. Nat. Rev. Microbiol..

[85] Cordeiro R.D.A., Evangelista A.J.D.J., Serpa R., Marques F.J.D.F., de Melo C.V.S., de Oliveira J.S., Franco J.D.S., de Alencar L.P., Bandeira T.D.J.P.G., Brilhante R.S.N. (2016). Inhibition of heat-shock protein 90 enhances the susceptibility to antifungals and reduces the virulence of Cryptococcus neoformans/Cryptococcus gattii species complex. Microbiology (Read. Engl.).

[86] Dorion S., Landry J. (2002). Activation of the mitogen-activated protein kinase pathways by heat shock. Cell Stress Chaperones.

[87] Nathan D.F., Vos M.H., Lindquist S. (1997). In vivo functions of the Saccharomyces cerevisiae Hsp90 chaperone. Proc. Natl. Acad. Sci. USA.

[88] Tiwari S., Thakur R., Shankar J. (2015). Role of Heat-Shock Proteins in Cellular Function and in the Biology of Fungi. Biotechnol. Res. Int..

[89] Chatterjee S., Tatu U. (2017). Heat shock protein 90 localizes to the surface and augments virulence factors of Cryptococcus neoformans. PLoS Negl. Trop. Dis..

[90] Perfect J.R. (2006). Cryptococcus neoformans: The yeast that likes it hot. FEMS Yeast Res..

[91] Kakeya H., Udono H., Maesaki S., Sasaki E., Kawamura S., Hossain M.A., Yamamoto Y., Sawai T., Fukuda M., Mitsutake K. (1999). Heat shock protein 70 (hsp70) as a major target of the antibody response in patients with pulmonary cryptococcosis. Clin. Exp. Immunol..

[92] Steen B.R., Lian T., Zuyderduyn S., MacDonald W.K., Marra M., Jones S.J.M., Kronstad J.W. (2002). Temperature-regulated transcription in the pathogenic fungus Cryptococcus neoformans. Genome Res..

[93] Soil D.R. (1997). Gene regulation during high-frequency switching in Candida albicans. Microbiology.

[94] Jacobson E.S., Jenkins N.D., Todd J.M. (1994). Relationship between superoxide dismutase and melanin in a pathogenic fungus. Infect. Immun..

[95] Wang P., Cardenas M.E., Cox G.M., Perfect J.R., Heitman J. (2001). Two cyclophilin homologs with shared and distinct functions important for growth and virulence of Cryptococcus neoformans. EMBO Rep..

[96] Li H., Yao Z., Degenhardt B., Teper G., Papadopoulos V. (2001). Cholesterol binding at the cholesterol recognition/ interaction amino acid consensus (CRAC) of the peripheral-type benzodiazepine receptor and inhibition of steroidogenesis by an HIV TAT-CRAC peptide. Proc. Natl. Acad. Sci. USA.

[97] Lee T.-H., Hirst D.J., Aguilar M.-I. (2015). New insights into the molecular mechanisms of biomembrane structural changes and interactions by optical biosensor technology. Biochim. Biophys. Acta.

[98] Srivatsav A.T., Mishra M., Kapoor S. (2018). Small-Molecule Modulation of Lipid-Dependent Cellular Processes against Cancer: Fats on the Gunpoint. BioMed Res. Int..

[99] Vincent V.L., Klig L.S. (1995). Unusual effect of myo-inositol on phospholipid biosynthesis in Cryptococcus neoformans. Microbiology (Read. Engl.).

[100] Luberto C., Toffaletti D.L., Wills E.A., Tucker S.C., Casadevall A., Perfect J.R. (2001). Roles for Iinositol-phosphoryl ceramide synthase 1 (IPC1) in pathogenesis of C. neoformans. Genes Dev..

[101] D’Souza C.A., Alspaugh J.A., Yue C., Harashima T., Cox G.M., Perfect J.R., Heitman J. (2001). Cyclic AMP-dependent protein kinase controls virulence of the fungal pathogen Cryptococcus neoformans. Mol. Cell. Biol..

[102] Sia R.A., Lengeler K.B., Heitman J. (2000). Diploid strains of the pathogenic basidiomycete Cryptococcus neoformans are thermally dimorphic. Fungal Genet. Biol..

[103] Kassi F.K., Bellet V., Drakulovski P., Krasteva D., Roger F., Valérie B.-T.A., Aboubakar T., Doumbia A., Kouakou G.A., Delaporte E. (2018). Comparative typing analyses of clinical and environmental strains of the Cryptococcus neoformans/Cryptococcus gattii species complex from Ivory Coast. J. Med. Microbiol..

[104] Soltani M., Bayat M., Hashemi S.J., Zia M., Pestechian N. (2013). Isolation of Cryptococcus neoformans and other opportunistic fungi from pigeon droppings. J. Res. Med. Sci..

[105] Bose I., Reese A.J., Ory J.J., Janbon G., Doering T.L. (2003). A yeast under cover: The capsule of Cryptococcus neoformans. Eukaryot. Cell.

[106] Denham S.T., Brown J.C.S. (2018). Mechanisms of Pulmonary Escape and Dissemination by Cryptococcus neoformans. J. Fungi.

[107] Gerwien F., Skrahina V., Kasper L., Hube B., Brunke S. (2018). Metals in fungal virulence. FEMS Microbiol. Rev..

[108] León-Sicairos N., Reyes-López M., Canizalez-Román A., Bermúdez-Cruz R.M., Serrano-Luna J., Arroyo R., de La Garza M. (2005). Human hololactoferrin: Endocytosis and use as an iron source by the parasite Entamoeba histolytica. Microbiology (Read. Engl.).

[109] De Leon-Rodriguez C.M., Fu M.S., Çorbali M.O., Cordero R.J.B., Casadevall A. (2018). The Capsule of Cryptococcus neoformans Modulates Phagosomal pH through Its Acid-Base Properties. mSphere.

[110] Fu M.S., Coelho C., de Leon-Rodriguez C.M., Rossi D.C.P., Camacho E., Jung E.H., Kulkarni M., Casadevall A. (2018). Cryptococcus neoformans urease affects the outcome of intracellular pathogenesis by modulating phagolysosomal pH. PLoS Pathog..

[111] Feder V., Kmetzsch L., Staats C.C., Vidal-Figueiredo N., Ligabue-Braun R., Carlini C.R., Vainstein M.H. (2015). Cryptococcus gattii urease as a virulence factor and the relevance of enzymatic activity in cryptococcosis pathogenesis. FEBS J..

[112] Casadevall A., Pirofski L.-A. (2007). Accidental virulence, cryptic pathogenesis, martians, lost hosts, and the pathogenicity of environmental microbes. Eukaryot. Cell.

[113] Park Y.-D., Williamson P.R. (2015). Masking the Pathogen: Evolutionary Strategies of Fungi and Their Bacterial Counterparts. J. Fungi.

[114] Zarrin M., Jorfi M., Amirrajab N., Rostami M. (2010). Isolation of Cryptococcus neoformans from pigeon droppings in Ahwaz, Iran. Turk. J. Med. Sci..

[115] Sorrell T.C., Ellis D.H. (1997). Ecology of Cryptococcus neoformans. Rev. Iberoam. Micol..

[116] Lee S.C., Phadke S., Sun S., Heitman J. (2012). Pseudohyphal growth of Cryptococcus neoformans is a reversible dimorphic transition in response to ammonium that requires Amt1 and Amt2 ammonium permeases. Eukaryot. Cell.

[117] Botts M.R., Hull C.M. (2010). Dueling in the lung: How Cryptococcus spores race the host for survival. Curr. Opin. Microbiol..

[118] Caza M., Kronstad J.W. (2019). The cAMP/Protein Kinase a Pathway Regulates Virulence and Adaptation to Host Conditions in Cryptococcus neoformans. Front. Cell. Infect. Microbiol..

[119] Velagapudi R., Hsueh Y.-P., Geunes-Boyer S., Wright J.R., Heitman J. (2009). Spores as infectious propagules of Cryptococcus neoformans. Infect. Immun..

[120] Feretzaki M., Heitman J. (2013). Genetic circuits that govern bisexual and unisexual reproduction in Cryptococcus neoformans. PLoS Genet..

[121] Hsueh Y.P., Xue C., Heitman J. (2007). G protein signaling governing cell fate decisions involves opposing alpha subunits in Cryptococcus neoformans. Mol. Biol. Cell.

[122] Dromer F., Mathoulin-Pélissier S., Launay O., Lortholary O. (2007). Determinants of disease presentation and outcome during cryptococcosis: The CryptoA/D study. PLoS Med..

[123] Casali A.K., Goulart L., Rosa e Silva L.K., Ribeiro A.M., Amaral A.A., Alves S.H., Schrank A., Meyer W., Vainstein M.H. (2003). Molecular typing of clinical and environmental Cryptococcus neoformans isolates in the Brazilian state Rio Grande do Sul. FEMS Yeast Res..

[124] Kassi F.K., Bellet V., Doumbia A., Krasteva D., Drakulovski P., Kouakou G.A., Gatchitch F., Delaporte E., Reynes J., Mallié M. (2016). First case of mixed infection with Cryptococcus deuterogattii and Cryptococcus neoformans VNI in an Ivorian HIV-positive patient. JMM Case Rep..

[125] Siddiqui R., Khan N.A. (2012). Acanthamoeba is an evolutionary ancestor of macrophages: A myth or reality?. Exp. Parasitol..

[126] Hurst J.K. (2012). What really happens in the neutrophil phagosome?. Free Radic. Biol. Med..

[127] Winterbourn C.C., Kettle A.J. (2013). Redox reactions and microbial killing in the neutrophil phagosome. Antioxid. Redox Signal..

[128] Leippe M., Herbst R. (2004). Ancient weapons for attack and defense: The pore-forming polypeptides of pathogenic enteric and free-living amoeboid protozoa. J. Eukaryot. Microbiol..

[129] Medina G., Flores-Martin S., Fonseca B., Otth C., Fernandez H. (2014). Mechanisms associated with phagocytosis of Arcobacter butzleri by Acanthamoeba castellanii. Parasitol. Res..

[130] Herbst R., Ott C., Jacobs T., Marti T., Marciano-Cabral F., Leippe M. (2002). Pore-forming polypeptides of the pathogenic protozoon Naegleria fowleri. J. Biol. Chem..

[131] Okagaki L.H., Strain A.K., Nielsen J.N., Charlier C., Baltes N.J., Chrétien F., Heitman J., Dromer F., Nielsen K. (2010). Cryptococcal cell morphology affects host cell interactions and pathogenicity. PLoS Pathog..

[132] Zaragoza O. (2011). Multiple Disguises for the Same Party: The Concepts of Morphogenesis and Phenotypic Variations in Cryptococcus neoformans. Front. Microbiol..

[133] Madu U.L., Ogundeji A.O., Pohl C.H., Albertyn J., Sebolai O.M. (2017). Elucidation of the Role of 3-Hydroxy Fatty Acids in Cryptococcus-amoeba Interactions. Front. Microbiol..

[134] van Oss C.J., Gillman C.F. (1974). Opsonic properties of human serum alpha-2 hs glycoprotein. Immunol. Commun..

[135] Jersmann H.P.A., Dransfield I., Hart S.P. (2003). Fetuin/alpha2-HS glycoprotein enhances phagocytosis of apoptotic cells and macropinocytosis by human macrophages. Clin. Sci..

[136] Sebolai O.M., Pohl C.H., Botes P.J., van Wyk P.W.J., Kock J.L.F. (2008). The influence of acetylsalicylic acid on oxylipin migration in Cryptococcus neoformans var. neoformans UOFS Y-1378. Can. J. Microbiol..

[137] Steenbergen J.N., Casadevall A. (2003). The origin and maintenance of virulence for the human pathogenic fungus Cryptococcus neoformans. Microbes Infect..

[138] Chrisman C.J., Albuquerque P., Guimaraes A.J., Nieves E., Casadevall A. (2011). Phospholipids trigger Cryptococcus neoformans capsular enlargement during interactions with amoebae and macrophages. PLoS Pathog..

[139] Trevijano-Contador N., Rossi S.A., Alves E., Landín-Ferreiroa S., Zaragoza O. (2017). Capsule Enlargement in Cryptococcus neoformans is Dependent on Mitochondrial Activity. Front. Microbiol..

[140] Janbon G. (2004). Cryptococcus neoformans capsule biosynthesis and regulation. FEMS Yeast Res..

[141] Zaragoza O., Rodrigues M.L., de Jesus M., Frases S., Dadachova E., Casadevall A. (2009). The capsule of the fungal pathogen Cryptococcus neoformans. Adv. Appl. Microbiol..

[142] Dambuza I.M., Drake T., Chapuis A., Zhou X., Correia J., Taylor-Smith L., LeGrave N., Rasmussen T., Fisher M.C., Bicanic T. (2018). The Cryptococcus neoformans Titan cell is an inducible and regulated morphotype underlying pathogenesis. PLoS Pathog..

[143] Granger D.L., Perfect J.R., Durack D.T. (1985). Virulence of Cryptococcus neoformans. Regulation of capsule synthesis by carbon dioxide. J. Clin. Investig..

[144] Okagaki L.H., Nielsen K. (2012). Titan cells confer protection from phagocytosis in Cryptococcus neoformans infections. Eukaryot. Cell.

[145] García-Rodas R., Zaragoza O. (2012). Catch me if you can: Phagocytosis and killing avoidance by Cryptococcus neoformans. FEMS Immunol. Med. Microbiol..

[146] Coelho C., Bocca A.L., Casadevall A. (2014). The intracellular life of Cryptococcus neoformans. Annu. Rev. Pathol..

[147] Steenbergen J.N., Shuman H.A., Casadevall A. (2001). Cryptococcus neoformans interactions with amoebae suggest an explanation for its virulence and intracellular pathogenic strategy in macrophages. Proc. Natl. Acad. Sci. USA.

[148] Brown G.D. (2011). Innate antifungal immunity: The key role of phagocytes. Annu. Rev. Immunol..

[149] Willment J.A., Brown G.D. (2008). C-type lectin receptors in antifungal immunity. Trends Microbiol..

[150] Cornillon S., Gebbie L., Benghezal M., Nair P., Keller S., Wehrle-Haller B., Charette S.J., Brückert F., Letourneur F., Cosson P. (2006). An adhesion molecule in free-living Dictyostelium amoebae with integrin beta features. EMBO Rep..

[151] Luberto C., Martinez-Mariño B., Taraskiewicz D., Bolaños B., Chitano P., Toffaletti D.L., Cox G.M., Perfect J.R., Hannun Y.A., Balish E. (2003). Identification of App1 as a regulator of phagocytosis and virulence of Cryptococcus neoformans. J. Clin. Investig..

[152] Williams V., Del Poeta M. (2011). Role of glucose in the expression of Cryptococcus neoformans antiphagocytic protein 1, App1. Eukaryot. Cell.

[153] Tan H.-Y., Wang N., Li S., Hong M., Wang X., Feng Y. (2016). The Reactive Oxygen Species in Macrophage Polarization: Reflecting its Dual Role in Progression and Treatment of Human Diseases. Oxidative Med. Cell. Longev..

[154] Paiva C.N., Bozza M.T. (2014). Are reactive oxygen species always detrimental to pathogens?. Antioxid. Redox Signal..

[155] Day B.J. (2009). Catalase and glutathione peroxidase mimics. Biochem. Pharmacol..

[156] Narasipura S.D., Chaturvedi V., Chaturvedi S. (2005). Characterization of Cryptococcus neoformans variety gattii SOD2 reveals distinct roles of the two superoxide dismutases in fungal biology and virulence. Mol. Microbiol..

[157] Rutkowski R., Pancewicz S.A., Rutkowski K., Rutkowska J. (2007). Reactive oxygen and nitrogen species in inflammatory process. Pol. Merkur. Lek..

[158] Naslund P.K., Miller W.C., Granger D.L. (1995). Cryptococcus neoformans fails to induce nitric oxide synthase in primed murine macrophage-like cells. Infect. Immun..

[159] Rosas A.L., Nosanchuk J.D., Feldmesser M., Cox G.M., McDade H.C., Casadevall A. (2000). Synthesis of polymerized melanin by Cryptococcus neoformans in infected rodents. Infect. Immun..

[160] Rodrigues M.L., Alviano C.S., Travassos L.R. (1999). Pathogenicity of Cryptococcus neoformans: Virulence factors and immunological mechanisms. Microbes Infect..

[161] De Jesús-Berríos M., Liu L., Nussbaum J.C., Cox G.M., Stamler J.S., Heitman J. (2003). Enzymes that counteract nitrosative stress promote fungal virulence. Curr. Biol..

[162] Noverr M.C., Phare S.M., Toews G.B., Coffey M.J., Huffnagle G.B. (2001). Pthogenic yeasts Cryptococcus neoformans and Candida albicans produce immunomodulatory prostaglandins. Infect. Immun..

